# From Flashes to Edges to Objects: Recovery of Local Edge Fragments Initiates Spatiotemporal Boundary Formation

**DOI:** 10.3389/fpsyg.2016.00910

**Published:** 2016-06-28

**Authors:** Gennady Erlikhman, Philip J. Kellman

**Affiliations:** Department of Psychology, University of California, Los AngelesLos Angeles, CA, USA

**Keywords:** illusory contours, perceptual organization, apparent motion, spatiotemporal integration, boundary formation

## Abstract

Spatiotemporal boundary formation (SBF) is the perception of illusory boundaries, global form, and global motion from spatially and temporally sparse transformations of texture elements (Shipley and Kellman, 1993a, 1994; Erlikhman and Kellman, 2015). It has been theorized that the visual system uses positions and times of element transformations to extract local oriented edge fragments, which then connect by known interpolation processes to produce larger contours and shapes in SBF. To test this theory, we created a novel display consisting of a sawtooth arrangement of elements that disappeared and reappeared sequentially. Although apparent motion along the sawtooth would be expected, with appropriate spacing and timing, the resulting percept was of a larger, moving, illusory bar. This display approximates the minimal conditions for visual perception of an oriented edge fragment from spatiotemporal information and confirms that such events may be initiating conditions in SBF. Using converging objective and subjective methods, experiments showed that edge formation in these displays was subject to a temporal integration constraint of ~80 ms between element disappearances. The experiments provide clear support for models of SBF that begin with extraction of local edge fragments, and they identify minimal conditions required for this process. We conjecture that these results reveal a link between spatiotemporal object perception and basic visual filtering. Motion energy filters have usually been studied with orientation given spatially by luminance contrast. When orientation is not given in static frames, these same motion energy filters serve as spatiotemporal edge filters, yielding local orientation from discrete element transformations over time. As numerous filters of different characteristic orientations and scales may respond to any simple SBF stimulus, we discuss the aperture and ambiguity problems that accompany this conjecture and how they might be resolved by the visual system.

## Introduction

**Figure 1:**
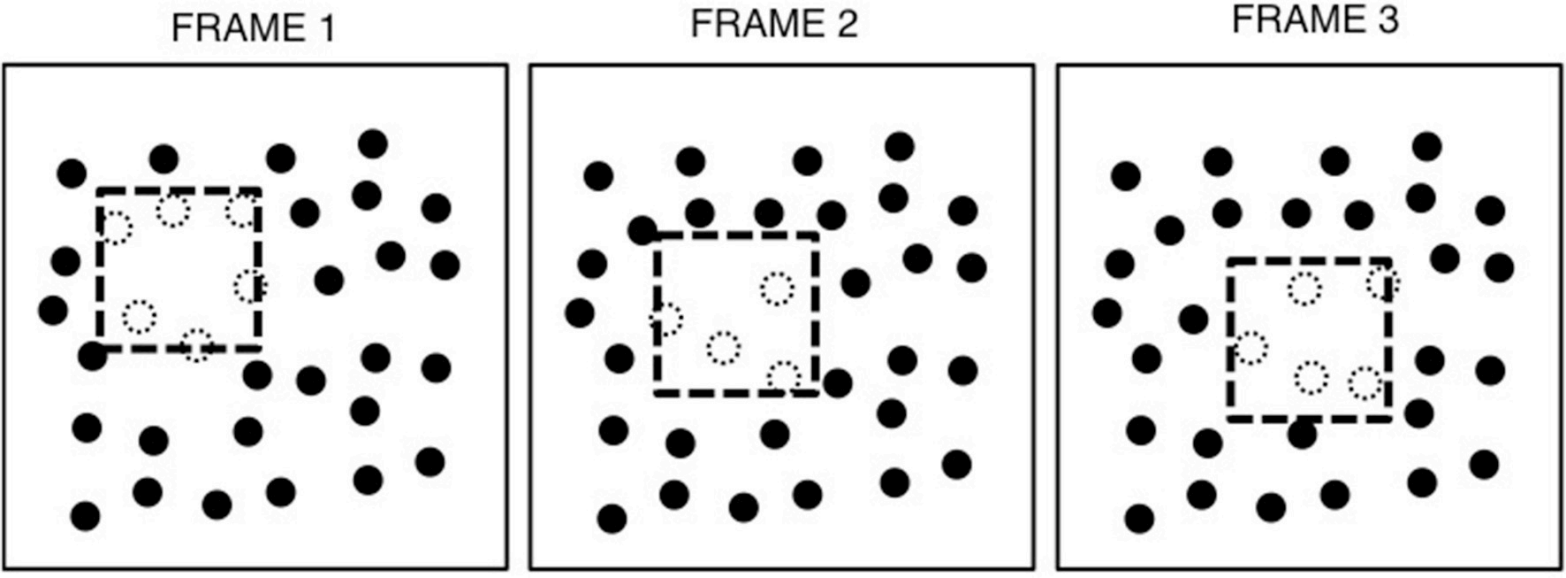
**An example of three frames from a typical SBF display**. The virtual object, a square, is indicated by the dashed line. Elements inside the object boundary are white, indicating that they share some surface property (e.g., color, orientation, shape), while those outside are black, having a different value for that property (e.g., red circles inside the boundary and green outside). As the virtual object moves, elements entering the boundary of the square become white and those exiting become black. Figure from Erlikhman and Kellman (2015).

Spatiotemporal boundary formation (SBF) is the perception of continuous contours, global form, and global motion from the sequential transformation of sparse texture elements (Shipley and Kellman, 1993a, 1994; Erlikhman and Kellman, 2015). An example of this process is shown in Figure 1, and the effect can be seen in Movie 1. In this figure, a virtual square is depicted as moving across a texture field. The object is virtual in the sense that its boundaries are not defined by luminance differences with the background. As the square moves, elements fall within or outside of its boundary. Upon entering or exiting the boundary, the elements change in some property, such as shape, orientation, or, as in this case color—from black to white when inside the boundary and vice versa when falling outside. This produces a pattern of element transformations along the boundary of the object as it moves across the display. The resulting percept is of clear, continuous illusory contours that correspond to the virtual object's boundary and, in this case, a perception of an internal surface. SBF is supported by other kinds of element transformations and does not require a distinct region in which all elements share the same property. For example, illusory contours are also seen if all elements are randomly oriented rectangles that change in their orientation upon entering or exiting the virtual object boundary. On a single frame from such a display, no clear object region is visible. Accretion and deletion of texture (Gibson et al., 1969; Kaplan, 1969; Andersen and Cortese, 1989) is an example of transformations that produce SBF, but the class of possible element transformations that produce perception of continuous contours, shape and motion is much broader (Shipley and Kellman, 1994).

Functionally, SBF may exist as a visual mechanism for apprehending objects under conditions of minimal information, as when the surface properties of two objects are similar or difficult to discriminate, or when an object is viewed under complex occlusion situations (e.g., through foliage), or under dim viewing conditions when surface features are difficult to resolve. SBF is perhaps the most extreme case in which the visual system constructs contours and objects from fragmentary input, as it requires no oriented edge fragments and produces complete perceived boundaries with little stimulus support. In normal, static, illusory contour figures, robust contour interpolation occurs with support ratios (the ratio of illusory or occluded edge length to total edge length) of 0.5 or greater, and noticeable interpolation may still be present at support ratios of 0.2 or 0.3 (Banton and Levi, 1992; Shipley and Kellman, 1992). In contrast, in SBF displays with widely spaced, small background elements, support ratio would be very close to zero, yet robust perception of continuous contours and clear overall shape are present (Shipley and Kellman, 1994).

How are shapes seen in SBF? One hypothesis is that the visual system somehow acquires local edge fragments from transformations of texture elements (e.g., occlusion) that occur closely together in space and time. These edges fragments, once created, connect across gaps to form concrete objects. Shipley and Kellman (1994, 1997) proposed a model of how edge orientation can be computed from the positions of elements, the distances between them, and the temporal interval between their transformations. This model has been shown to fit human data very well across a number of experiments (Erlikhman and Kellman, 2015). In separate experiments, several constraints on this process have been identified. First, element transformations must be encoded as discontinuities or disruptions in the regular pattern of the textured background (Shipley and Kellman, 1993a). If changes are perceptually detectable, but small (e.g., a small displacement of each element as it enters or exists the boundary), then no illusory shape is seen. Instead, such a display is seen as a surface with some non-rigid jiggling. Most detectable element transformations including color changes, element rotation, and position changes support SBF, except for equiluminant color changes (Miyahara and Cicerone, 1997). Second, at frame durations longer than ~165 ms, illusory contours, global form, and global motion are no longer seen (Shipley and Kellman, 1994). Third, SBF degrades with decreasing texture density. However, texture density confounds spatial and temporal distances between transformation events as well as the total number of element transformations that occur. Density also interacts with contour complexity such that spatially sparse textures will result in fewer interactions with the virtual object border so that high curvature regions will not interact with as many elements as regions with lower curvature. A study in which the arrangement of texture elements was not random, also found that boundaries were more clearly seen for higher densities in which there were smaller gaps between elements (Fidopiastis et al., 2000). When no global form was seen, observers reported seeing element-to-element apparent motion between transforming elements.

Despite these efforts to characterize and model the SBF process, it has been difficult to isolate the precise spatial and temporal constraints. Because virtually all experiments have used large texture arrays and two-dimensional virtual shapes, it has been impossible to control display properties such as inter-element distance and the timing between element transformations simultaneously (although see Fidopiastis et al., 2000). Furthermore, the use of closed shapes as the virtual objects in SBF experiments necessarily entangles the two proposed stages of the model: construction of local edge fragments from element transformation events followed by contour interpolation of those fragments into a complete shape. It is therefore unknown whether SBF operates over small or large distances, whether the previous findings regarding density and timing are constraints on the local edge formation process or on a secondary stage that integrates those edges into a global form, and it is also unknown what the minimal conditions are for seeing the simplest possible SBF-defined figure: a single edge.

The present experiments set out to address these questions. First, we sought to study displays that arguably approached the minimum conditions for seeing any local oriented contour fragment in SBF. The idea of two stages in SBF, the first being some process that establishes local, oriented contour fragments, is theoretical; based on existing data, it is possible that SBF does not even occur in the absence of closed 2D forms or long extended contours. Second, we wished to study display conditions in which absolute spatial and temporal distances between element transformation events as well as the total number of events that occurred per frame could be controlled and manipulated. Precise measurements of spatiotemporal integration limits are important for modeling SBF and they also allow for a more ready comparison of SBF to other, well-characterized visual phenomena in which elements transform successively, such as apparent motion. Second, using these displays, a specific prediction of the proposed SBF model could be tested: that a single, illusory edge fragment can be perceived from a small number of successive, non-simultaneous, element transformations. This prediction is surprising and unexpected because sequential disappearances and reappearances of elements are precisely the conditions under which either apparent or phi motion are seen (Wertheimer, 1912). No motion models instead predict the perception of illusory contours; within certain spatial and temporal intervals, they would predict apparent motion from the location of one element change to the next one in succession. Indeed, this ordinary percept of apparent motion between element transformations is seen when SBF breaks down and does not produce perception of contours and forms (Shipley and Kellman, 1993a).

## Experiment 1

**Figure 2:**
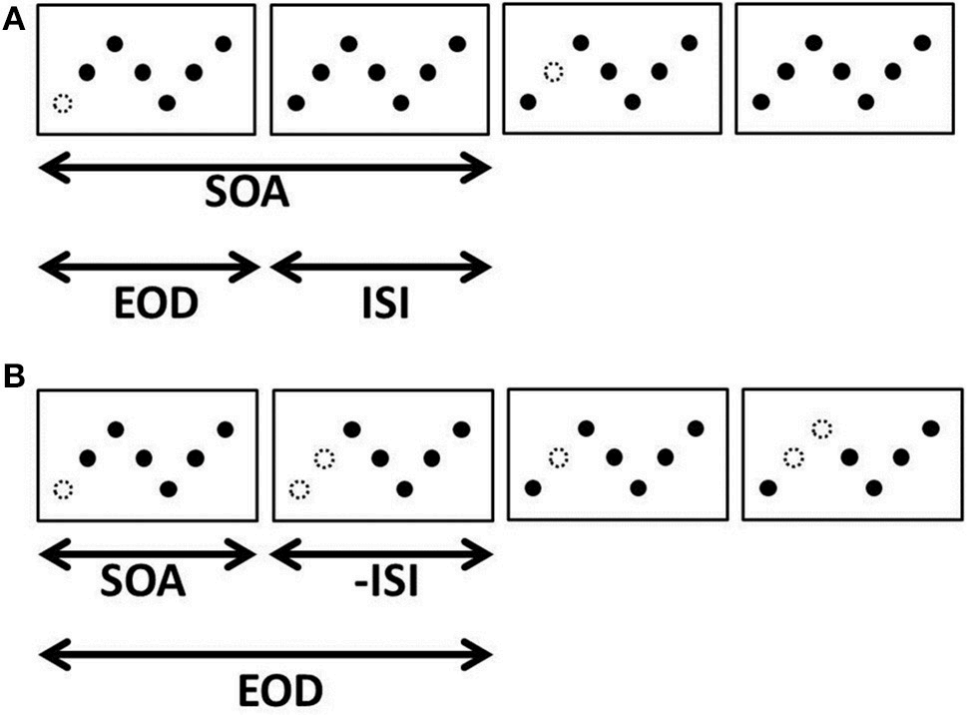
**Four apparent motion frames in which elements disappear one at a time in sequence**. We refer to the duration that an element is invisible as the element offset duration (EOD), the temporal interval between element disappearances as the inter-stimulus interval (ISI), and the total time between the disappearance of one element and the disappearance of the next as the stimulus onset asynchrony (SOA). **(A)** A case where a single element disappears per frame and reappears before the next disappears. **(B)** A case in which a second element disappears before the first reappears. In this example, EOD is longer than SOA so ISI is negative.

Consider a sawtooth arrangement of dots in which dots disappear and reappear one at a time in sequence (Figure 2A). What might one expect to see in such a display? For many spatial and temporal intervals, the laws governing apparent motion would predict that we should see a white dot or blob moving along the sawtooth pattern (Wertheimer, 1912; Korte, 1915; Ekroll et al., 2008). Indeed, this is exactly what is seen in Movie 2. In this movie, all elements are visible for 80 ms; one element disappears for 40 ms; all elements are again visible for 80 ms, etc. These settings are well within the range when apparent or phi-motion should be seen (Wertheimer, 1912; Steinman et al., 2000; Ekroll et al., 2008).

**Figure 3:**
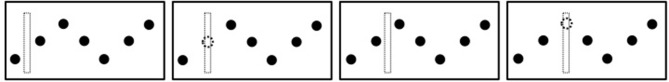
**The same display as in Figure 2**. Element disappearances (changes to white) are triggered by the passing of a virtual bar, indicated by the dashed rectangle.

Although the display depicted in Figure 2 has a perfectly valid physical description as a moving white dot that successively occludes a series of black dots, there is another description that characterizes this display as an SBF stimulus. The same series of transformations can also be the result of a white bar successively occluding each element (Figure 3). Under certain spatial and temporal conditions, it is indeed possible to see a moving white bar (Movie 3). The only difference between Movies 2, 3 is that the temporal interval during which all elements were visible has been shortened in Movie 2, from 80 to 0 ms. This change corresponds to shortening both the SOA and ISI to 40 and 0 ms, respectively, while keeping element offset duration (EOD) fixed. It should be noted that although the display is described in terms of an occluding bar, the elements are disappearing and reappearing discretely—there is no gradual occlusion or straight edges anywhere in the display.

Before considering why edges are seen in these displays and fully describing the conditions under which they are seen, we sought to demonstrate that it is possible to systematically generate displays in which either apparent motion or SBF are seen, and, importantly, that the resulting percepts are robust and consistent across observers. In order to do so, observers viewed a set of SBF movies in which the velocity and width of the illusory bars was manipulated. We used both an objective performance task and a subjective report task using the same observers. Observers made forced-choice judgments of perceived bar width and provided ratings of illusory contour strength.

### Materials and methods

#### Participants

Subjects were five research assistants or graduate students (one of whom was one of the authors, GE) who volunteered for the study (4 female; age range: 21–27). All subjects reported having normal or corrected-to-normal vision. All subjects except for the author were naïve to the purposes of the study, but were highly trained psychophysical observers. Experiments were approved and conducted under the guidelines of the UCLA IRB. All experiments followed the Declaration of Helsinki guidelines. All subjects provided informed consent to participate.

#### Apparatus

All displays were created and displayed using the MATLAB programming language and the Psychophysics Toolbox (Brainard, 1997; Pelli, 1997). Stimuli were presented on a Viewsonic G250 CRT monitor, which was powered by a MacPro 4 with a 2.66 GHz Quad-Core Intel Xeon processor and an NVidia GeForce GT120 graphics card. The monitor was set to a resolution of 1024 × 768 pixels and a refresh rate of 100 Hz.

#### Displays

Displays consisted of black dots (diameter = 7.9 arcmin) on a white background (13.42 × 10.08° of visual angle) arranged in a sawtooth pattern with five dots per cycle. The dots were centered on the screen vertically and spanned its entire width. The vertical and horizontal distances between dots (measured from their centers) were 0.4°. There were a total of 33 dots spanning the width of the screen.

There are two equivalent ways to describe the display transformations in the experiment. In all cases, the transformations consisted of sequential changes in the array, with one dot at a time disappearing (changing to match the white background) and later reappearing in the same position. Thus, the timing of these sequences of element offsets and onsets fully describes a given display. A second way to describe the displays, which we use here, both to connect with earlier work on SBF and to give an intuitive understanding of the objective performance method described below, is to describe dot disappearances in terms of a virtual bar moving across the array. In this study, the bar moved at a rate of either 2.6 or 13.2°/s. Whenever an element (dot) fell within the boundary of the bar, that element became invisible (white); all other elements remained black. Note that the discrete disappearance of dots in this experiment is not the gradual covering and uncovering that would occur with actual movement of an occluding object. Four bar widths were used: 0.13, 0.26, 0.39, and 0.53°. Each bar width was presented 15 times moving at each speed, for a total of 120 trials. Bars always began at the left-most side of the screen and moved across the screen. Once they reached the opposite end of the display, they reversed direction. Trial order was randomized.

Above the dot sawtooth, four black horizontal lines were drawn side-by-side, with the group centered horizontally above the display. The four lines corresponded to the four bar widths. Each line was labeled with a number above it (1–4) that corresponded to the response keys. Subjects performed a four-alternative forced choice (4AFC) task by matching the perceived bar width to one of the four choices. After they made their response, the four lines disappeared and subjects were presented with text in their place asking them to press a number key 1–7 to rate the illusory strength of the bar. Displays lasted until subjects responded to both questions.

#### Procedure

Subjects sat at a distance of 170 cm from the computer monitor with their heads stabilized by a chin rest. The only illumination in the room came from the monitor. Subjects were told that they would be performing two tasks: first, they would estimate the perceived bar width by comparing it to one of four options, and second, they would rate the illusory strength of the bar.

The logic of the objective performance task was as follows. When SBF occurs in these displays, a clear bar oriented perpendicularly to the long axis of the sawtooth array is seen. Pilot work suggested that such perceived bars have determinate width, and that their width is consistent with the width predicted by the moving virtual bar used to determine the element offsets and onsets. Under other spatial and temporal parameters for the dot onsets and offsets, however, SBF does not occur and thus there is no percept of bar width. We assumed that in the absence of a perceived bar, participants would be unlikely to give responses indicating the true width of the virtual bar. Note that both in cases under which SBF does and does not occur in this experiment, there is always an objectively correct answer about the virtual bar used to generate a given display. We consider this task to be an objective performance assessment of perception because a participant's judgments of bar width can be compared to a geometrically derived standard.

We also felt that it was important to directly assess participants' impressions of whether and when an illusory bar and attendant illusory contours occurred in these displays. We predicted that spatial and temporal parameters that would lead to clear illusory bar perception would also be those that produced accurate performance on the bar-width estimation task. We hypothesized that the presence or absence of SBF across various displays would be accessed both by the objective task and by subjective reports of illusory contour strength.

For the magnitude estimation (subjective report) task, a Kanizsa square was used as an example of a strong illusory contour, with participants being instructed that it was an example of a display corresponding to a rating of 7. A 1 rating corresponded to the absence of an illusory contour and was demonstrated by drawing attention to the rounded portions of the pacmen figures used to create the Kanizsa square and showing that no contour was perceived to connect the outer parts of the pacmen in any way. Subjects were then instructed that they would see several movies containing a sawtooth pattern of black dots on a white screen and in which one dot would disappear at a time. They were told that if they tracked the flashing pattern laterally, they would sometimes see a moving illusory bar. This instruction was important because from pilot testing it was observed that the illusion was weaker in the periphery and also when subjects fixated in the middle of the screen. Once subjects confirmed that they understood the instructions, they pressed a key to begin the experiment. Subjects first estimated the perceived width of the bar by pressing a key 1–4 on the keyboard corresponding to one of four lines shown on the display that represented the four possible bar widths. Subjects then provided a rating of illusory contour strength for the same display. After providing a rating, a blank, white screen was shown for 500 ms and then the next trial began immediately. There was no time limit for either question in a trial. No breaks were provided, although subjects were instructed that if they were feeling fatigued or experiencing eye strain, they could rest on any trial since there was no time limit.

### Results

#### Objective task: perception of bar width

**Figure 4:**
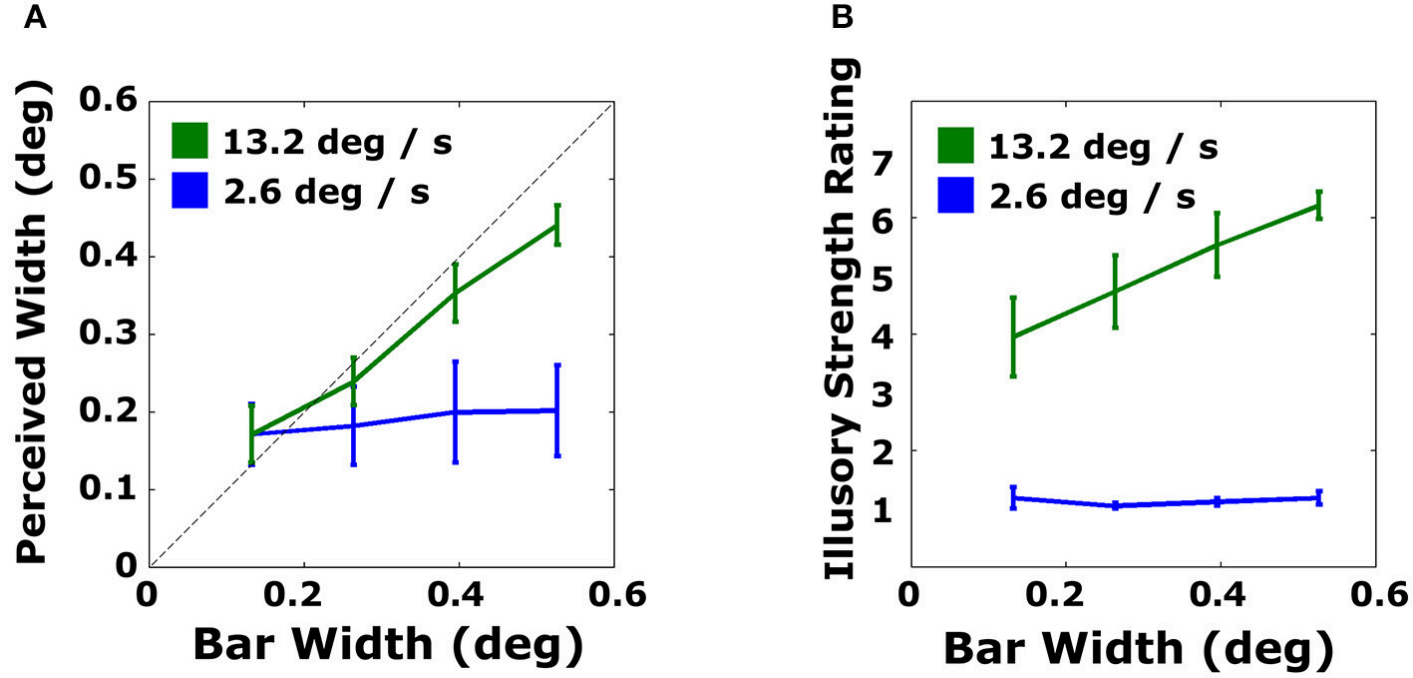
**Average performance in Experiment 1 for estimating perceived bar width (A) and illusory strength ratings (B) for slow (blue) and fast (green) bars**. The dashed line indicates veridical performance in the objective task. Error bars are standard errors.

Judged bar widths as a function of virtual bar widths are shown in Figure 4A. A striking difference can be seen for plots showing data for the faster and slower speed. For the slower speed, across all widths, participants showed no evidence of perceiving bar width; they appeared to default to giving ratings of the shortest width for all displays. Participants reported after the experiment that when they did not see a bar, most defaulted to selecting the shortest length. In contrast, subjects were able to perceive the width of faster bars (green lines) with much greater accuracy. There was a slight bias in perceiving the widest bars as narrower (0.44°) than their true width (0.53°).

The data were fit with best-fitting linear regressions. For the objective measure, slopes were 0.702 [*t*_(4)_ = 15.4, *p* < 0.005] and 0.083 [*t*_(4)_ = 1.91, *p* > 0.12] for the fast and slow bars, respectively. Linear regressions were also fit to individual subject data. For fast bars, fitted slopes ranged from 0.453 to 0.973 in the objective task. For slow bars, fitted slopes for perceived width as a function of actual bar width ranged from 0.020 to 0.247.

#### Subjective task: magnitude estimates of illusory contour strength

The subjective report data (Figure 4B) showed similar patterns to the data from the objective task. For the slower virtual bar velocity, participants gave consistently the lowest possible ratings for all virtual bar widths, indicating that illusory contours were not seen. In contrast, mean ratings for all virtual bar widths at the faster velocity were 4 or above on the 7-point scale. Illusory strength ratings increased with bar width at the faster speed.

### Discussion

This experiment demonstrated that single edge fragments can be recovered from the sequential disappearance and reappearance of individual elements. Subjective and objective measures converged in that perceived bar width was accurate when subjects reported seeing strong illusory contours. The percept could be easily controlled by simply changing the speed of the virtual object (the timing of sequential offsets and onsets of individual elements) without altering any other properties of the display. Based on both the objective performance and subjective rating tasks, SBF was seen for virtual bars moving at 13.2°/s, but not bars moving at 2.6°/s in this study. This “virtual velocity” difference affected temporal properties in the displays: EOD and ISI (how long all elements were visible) were both shorter for the faster displays. The results of this experiment indicate that establishing oriented edge fragments in SBF depends on temporal parameters, but the data permit a variety of hypotheses about which temporal properties, such as EOD or ISI, matter and what their limits might be. Moreover, all displays in Experiment 1 used fixed spacing, whereas it seems likely that relations of spacing and timing may be relevant for understanding spatiotemporal edge formation. We carried out Experiment 2 to investigate spatial and temporal parameters more thoroughly.

There are some other aspects of the temporal and spatial parameters that are worth mentioning at this point. Because the bars were oriented vertically, EOD and ISI were constant. However, SBF does not require either constant timing of transformation events nor illusory edges that are perpendicular to the direction of motion. Bar orientation can easily be manipulated simply by adjusting EOD and ISI. A tilted bar moving laterally across the same display will produce variable ISIs between elements, with shorter intervals between elements that lie on paths parallel to the bar and longer intervals between changes of elements that lie on paths perpendicular to the bar. An example of the same display but with an oriented edge can be seen in Movie 4.

For illusory edges to be seen in these displays, the elements must be arranged in a non-collinear pattern; the sawtooth array is a simple choice. If the elements were collinear, no edge would be seen. Indeed, with a line of dots one would see either apparent motion only. Why must elements be so arranged in order to the produce SBF? Shipley and Kellman (1994, 1997) demonstrated that the orientation of an edge can be unambiguously determined from the spacing and timing of at least three, non-collinear element transformations. For collinear elements, the timing of element transformations is not affected by edge orientation. This is a form of an aperture problem that occurs in SBF (Shipley and Kellman, 1994, 1997; Prophet et al., 2001).

It is worth emphasizing how surprising it is that by having one dot disappear at a time, one can generate robust percepts of illusory contours and surfaces. Furthermore, one can easily adjust the properties of the resulting figure like width, velocity, or orientation simply by changing the temporal properties of the display. Consider what happens as bar width increases: elements are invisible for a longer period of time (since a wider bar takes longer to pass over them) and the time between element disappearances (ISI) is shorter. However, only one element is hidden at a time for both short and long bars. If one looks at the frames of this sequence, there is no information in any single frame not only about the edges of this bar, but also about its width. When an element is invisible, for either a fat or thin bar, the display is exactly identical, but the illusory contours appear in different places!

## Experiment 2

Experiment 1 showed that local edge orientations can be evoked by sequential changes in small arrays of unmoving elements. It also showed that edge perception occurs with some spatiotemporal relationships of element changes but not with others. The results do not permit much specification of the spatial and temporal constraints on SBF, however. For example, velocity and timing parameters were confounded while the distance between dots was held constant. SBF might also occur at slower velocities if more events occurred during that time, i.e., if the dots were packed more closely together. The inter-element spacing in Experiment 1 was chosen by pilot testing and was known to produce SBF. However, a minimum or maximum inter-element separation might be necessary to support SBF. In Experiment 2, we parametrically varied velocity and bar width at the same time as element spacing. If the constraints on SBF are purely temporal in nature, we expected there to be no effect of manipulating element spacing, as long as a similar number of element transformations occurred within a given temporal window. If SBF is instead constrained by a spatial or spatiotemporal window, so that element transformations have to occur within a certain proximity and temporal interval, these properties would interact.

### Materials and methods

#### Participants

Subjects were five research assistants or graduate students (one of whom was one of the authors, GE) who volunteered for the study (2 female; age range: 23–28). All subjects reported having normal or corrected-to-normal vision. Two of the subjects had participated in Experiment 1 and the remaining three were naïve to the purposes of the experiment.

Experiments were approved and conducted under the guidelines of the UCLA IRB. All subjects provided informed consent to participate.

#### Displays, apparatus and procedure

The same apparatus and stimuli were used as in Experiment 1 except for the changes noted below. The same four bar widths were tested. Five bar velocities were used: 2.6, 5.3, 7.9, 10.5, and 13.2°/s. Ten inter-element spacings were tested from 0.2 to 2.0° in steps of 0.2°. All combinations of velocities, bar widths, and spacings were repeated 10 times for a total of 2000 trials. Trials order was randomized. Subjects completed four, 1-h sessions on separate days, completing 500 trials per session. As in Experiment 1, on each trial, subjects were asked to match the perceived bar width to one of four choices followed by a subjective rating of illusory contour strength. All other aspects of the experiment were similar to Experiment 1.

### Results and discussion

**Figure 5:**
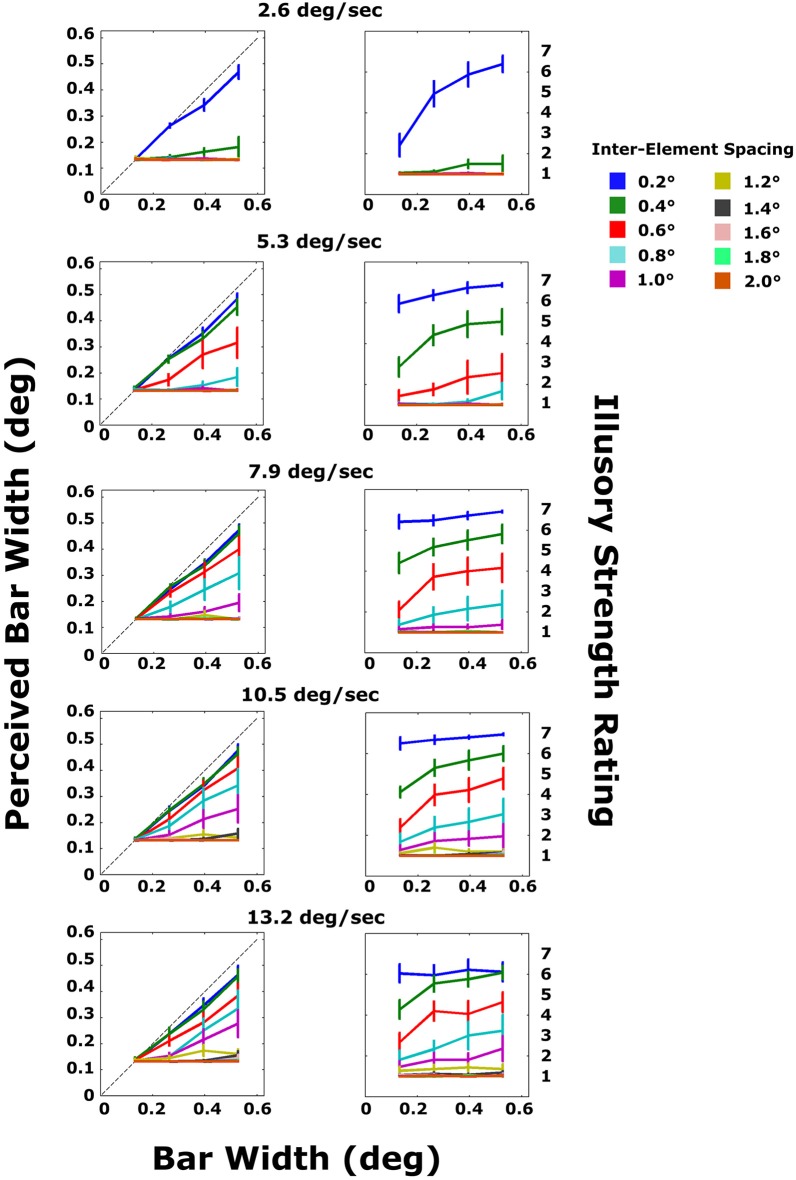
**Average subject data for the objective and subjective tasks in Experiment 2**. Each plot shows the results for a different bar velocity. Line colors correspond to different inter-element spacings. The dashed line in the top plots indicates veridical perception of bar width. Error bars are standard errors.

The results are shown in Figure 5. Data were split by velocity and inter-element spacing and averaged across subjects. Several interesting patterns emerged. First, for the smallest inter-element distance (0.2°; blue lines), subjects were able to accurately estimate bar width for all velocities (top panels), including the slowest velocity for which subjects were unable to do so in Experiment 1 (top-left panel). Second, the greater the velocity, the greater the maximum inter-element spacing for which bar widths could be accurately estimated. However, beyond an inter-element spacing of 1° (purple lines), no illusory contours could be seen for any velocity that was tested (lower panels). It cannot be determined, from this experiment alone, whether this is a hard spatial integration limit or whether the SBF can occur for larger inter-element spacings as velocity continues to increase. Third, as in Experiment 1, illusory contour strength ratings closely mirrored accurate estimation of bar width (see analysis below). Finally, a graded effect was observed, such that both objective performance and subjective ratings gradually decreased with increasing inter-element spacing at all velocities. The closer the spacing, the better the performance and the higher the ratings. See **Figure 7**.

**Figure 6:**
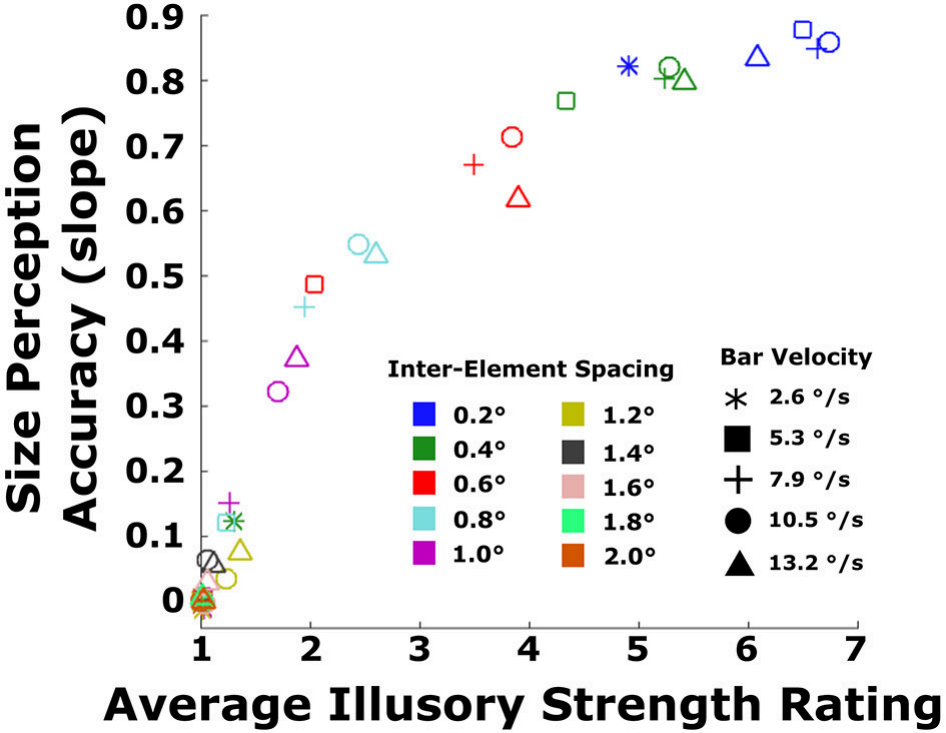
**Replotted data from Figure 5 depicting the size perception accuracy from the objective performance data plotted against subjective ratings, averaged across bar width**. Size perception accuracy is the slope of each line in the left column of panels in Figure 5. Data are shown for all 50 combinations of the five velocities (symbol shapes) and 10 inter-element spacings (symbol colors).

To better illustrate the relationship between objective performance and subjective ratings, the two measures were converted into scores that could easily be compared (Figure 6). As in Experiment 1, linear regressions were fitted to all data from the objective task, and the slope of each line was computed for each inter-element spacing and velocity. The slope characterized the size perception accuracy, with values closer to 1.0 indicating veridical perception of bar width and values closer to zero indicating no relationship between perceived and true width. This resulted in 50 slope values across all conditions. For the subjective ratings, the scores were summarized by taking the average of ratings across bar widths for each combination of velocity and inter-element spacing. For most conditions, with the exception of the smallest inter-element spacing and slowest velocity, ratings were relatively similar across bar widths for a given combination of velocity and spacing. This resulted in 50 average ratings across all velocity and spacing conditions. To assess quantitatively the concordance between the objective size perception data (given by slope) and the subjective ratings, we plotted the size perception accuracy (slopes) against the average ratings of illusory contour strength. There was a monotonic relationship between the two, with size perception accuracy increasing as a function of illusory strength rating (Spearman's rho = 0.893, *p* < 0.0001).

Because the bar moved at a constant velocity and because the timing was constant between element transformations (ISI and *f* were the same for every element), it was possible to compute the timing properties of the display from the physical properties of the bar stimulus using the following identity:

(1) $$\begin{array}{l} v=\frac{h}{SOA} \end{array}$$

In Equation (1), *v* is the object velocity and *h* is the horizontal separation between elements. Since *SOA* is defined as the sum of ISI and EOD (see Figure 2), any of these temporal terms can be substituted by a combination of the other two and the expression can be rewritten in terms of ISI and EOD. In order to distinguish between ISI and EOD, a second identity is needed. We take advantage of the fact that the time that an element is occluded (EOD) can also be interpreted as the time it takes for the bar to completely pass over an element. Given that elements disappeared and reappeared discretely with no gradual covering or uncovering, we treated the moment of element disappearance and reappearance as the point when the bar's edge reached (disappearance) or passed (reappearance) the element. The distance that the bar travels during the time that a dot it occludes is invisible (EOD) is therefore the bar's width, *w*:

(2) $$\begin{array}{l} w=v*EOD \end{array}$$

**Figure 7:**
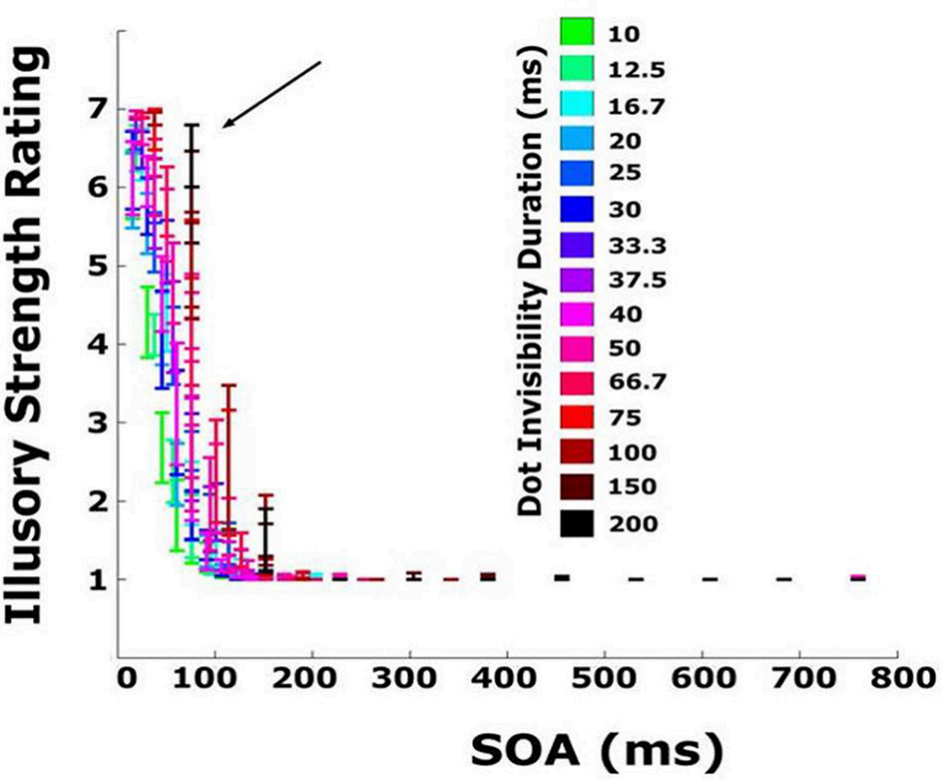
**Subjective rating data from Experiment 2 replotted as a function of SOA and EOD (time and element was invisible/occluded/white)**. Colors represent different EODs. For displays with small inter-element distance and slow, wide bars, several elements were occluded simultaneously, resulting in a EOD that was greater than the SOA. The black arrow indicates the 76 ms timepoint beyond which illusory strength ratings steeply decline.

Using Equations (1) and (2), it is possible to compute all three timing parameters for every display tested. The subjective rating data from Experiment 2 are replotted in Figure 7 as a function of SOA, separated by EOD.

**Figure 8:**
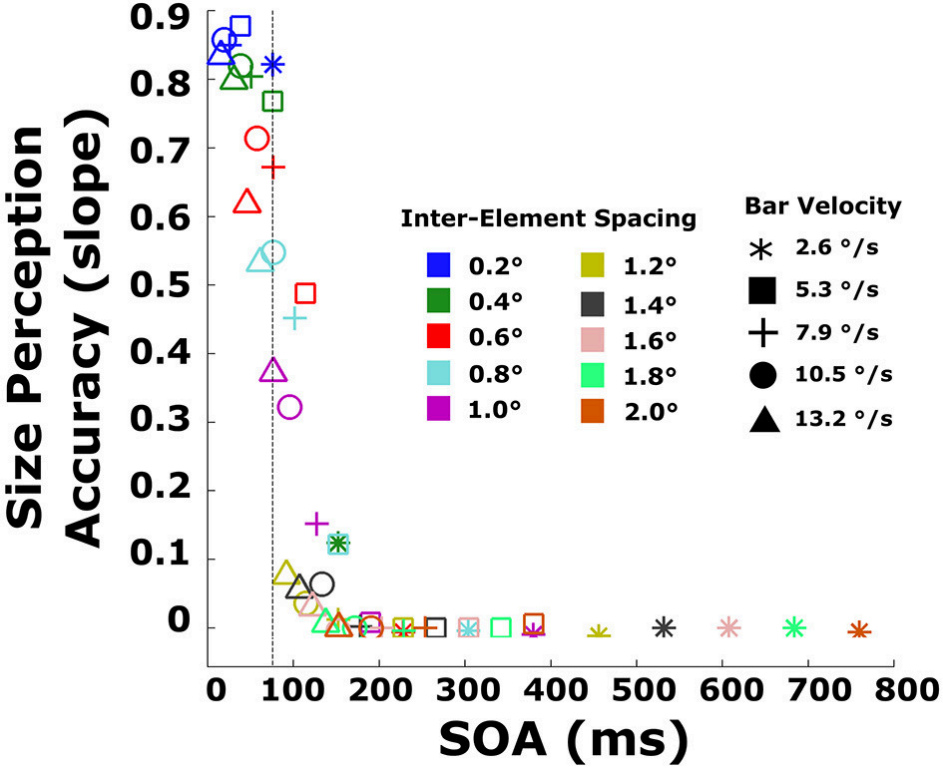
**Size perception accuracy (slopes of lines in the objective task) plotted against the SOA for every combination of velocity and inter-element spacing**. The dashed line indicates the 76 ms timepoint.

Several new patterns emerge. First, illusory strength declines sharply with increasing SOA. Beyond SOAs of ~100 ms, illusory contours are never seen irrespective of EOD. Second, for very short SOAs, below 40 ms, EOD has no effect on illusory contour strength. Third, at an SOA of 76 ms (marked by an arrow in the figure), illusory bars are seen for virtually all EODs, including the longest (200 ms). For this SOA, illusory contour strength increases as a function of EOD. A similar pattern can be observed for other SOAs between 40 and 76 ms. Note that the pattern of EOD appearing to increase as a function of SOA (bars go from green to black, left to right) is a function of how the displays are constructed: bars moving very quickly correspond to short EOD (pass over an element quickly) and small SOAs (reach the next element quickly), while slow bars correspond to long EOD and long SOAs. Note also that sometimes EOD exceeds SOA. This corresponds to displays in which the bar width was greater than the distance between elements so that a second element disappeared before the preceding element reappeared (Figure 2B). A similar pattern of results can be observed in the objective performance data (Figure 8). The 76 ms cutoff is marked for convenience with a dashed line. Size perception accuracy can be seen to decrease as a function of inter-element spacing and is independent of bar velocity for the smallest inter-element spacings. Importantly, size perception accuracy plummets very rapidly around the same cutoff.

This analysis reveals that SBF is limited by the timing between element transformations (SOA). Specifically, beyond an SOA of ~80 ms, edges cannot be extracted from such sparse element transformation events. An SOA-based limit explains why illusory contour strength ratings increased with bar width in both experiments: given a constant velocity, increasing bar width decreases the SOA. For very narrow bars, it takes longer to reach the next element. The limit also accounts for why ratings for the same inter-element spacing and bar widths increase as a function of velocity. Note, for example, that as velocity increases, ratings for the smallest inter-element separation (0.2°; blue lines) also increase, even for the shortest bar width. Increasing velocity reduces SOA in a similar manner to increasing bar width: the faster a bar is traveling, the shorter the time between element transformation events.

The temporal limit is also in agreement with previous attempts to identify temporal constraints of SBF. SBF models posit that a minimum of three non-collinear element transformations are needed to be able to extract edge orientation and velocity information (Shipley and Kellman, 1994, 1997; Erlikhman and Kellman, 2015). An SOA of 80 ms corresponds to a total time of 160 ms for three events to occur if the first event occurs at time 0. This matches closely to previous findings that shape identification in SBF displays improves with an increasing number of frames up to a total display duration of 150–165 ms, after which point additional frames do not contribute to performance (Shipley and Kellman, 1993b; Cunningham and Shipley, 2001). However, previous studies have exclusively used 2-dimensional, extended virtual objects and have not directly probed the maximum interval within which element transformation events can be integrated in more local formation of edge fragments. This experiment is the first to identify such limits.

## General discussion

Experiments 1 and 2 show that it is possible to recover individual edge fragments from minimal displays in which only a single element transforms per frame, and in which only a small, local, oriented edge is recovered. Thus, SBF is not limited to closed 2D figures. The experiments also revealed that properties of these edge fragments such as velocity, width, and orientation can be readily controlled by altering the duration of element disappearances and the timing between their disappearances. Finally, integration of element transformation events is constrained by clear temporal limits: Over a range of spacing and timing parameters, the controlling variable appears to be the occurrence of three element transformations within an ~165 ms window of integration (c.f., Shipley and Kellman, 1994; Cunningham and Shipley, 2001).

These findings offer new insight about the perception of contours and objects from sparse texture element changes. Most previous SBF displays had exclusively used 2D virtual objects (e.g., Shipley and Kellman, 1993a, 1994, 1997; Cicerone et al., 1995; Cunningham et al., 1998; Erlikhman et al., 2014; but see Chambeaud et al., 2014). The demonstration that SBF can occur for short edge fragments indicates that such fragments can be recovered without more global shape information and that such fragments are the likely basic units in SBF. This is in agreement with our current SBF models (Shipley and Kellman, 1994, 1997; Erlikhman and Kellman, 2015) and is inconsistent with others that rely on the detection of a large object region (Prophet et al., 2001). According to formal models (e.g., Shipley and Kellman, 1997), edge orientation and local motion can be extracted from the transformations of three, non-collinear elements given only their relative positions and the timing of the transformation events. In recent work based upon this account, we have shown that psychophysically obtained estimates of noise in the visual extraction of position and timing information accounts for variability in the perceived orientation of extracted edge fragments (Erlikhman and Kellman, 2015).

Once an edge fragment is extracted, it is connected to other fragments by interpolation processes that depend on their relative positions and orientations, as in more familiar phenomena such as perception of partly occluded objects and illusory contours (Kellman and Shipley, 1991; Palmer et al., 2006). The orientations and velocities of several differently oriented fragments can be used to determine the global motion direction of shapes defined by SBF (Shipley and Kellman, 1997). Because several events need to occur before an edge can be extracted, these fragments will never be available all at once. Rather, the visual system needs a method for maintaining a persisting representation of a fragment once extracted and updating its position relative to other fragments extracted at a later time (Palmer et al., 2006). Global form in SBF would then be constructed in the following manner: (1) local edge fragments are recovered from sequences of element transformations within a small spatiotemporal window, (2) relatable contour fragments are interpolated to produce a coherent boundary, (3) the global motion of the completed object is recovered from the individual motions of the edge fragments. Further work is needed to determine how many edge fragments are used to construct a complete object representation and how those edges are integrated. It may be the case, for instance, that (2) and (3) occur concurrently, or that the order is reversed. The present experiments cannot address this issue. In using only a single bar, we have introduced an aperture problem—its motion direction, although perceived to be horizontal across the display, is actually ambiguous, as it would be for any moving bar seen through an aperture.

An interesting property of the sawtooth displays is that, when the illusory bar is seen, both its trailing and leading edge are visible. The trailing edge is likely created by the same SBF process that determines the leading edge; however, the relevant element transformations for the trailing edge are the reappearances of dots rather than their disappearances.

It is surprising that no apparent motion is seen, especially when bars are moving quickly so that when one element disappears, the preceding element reappears on the same frame as in a typical apparent motion display. Correspondence models of apparent motion and first-order motion detectors would both predict that motion should be seen between elements (e.g., Ullman, 1979). Furthermore, the 80–100 ms limit matches the inter-frame interval beyond which apparent motion is not seen in random dot kinematograms (Baker and Braddick, 1985). However, a number of studies have found that form perception can alter or suppress motion perception (Petersik and McDill, 1981; Ramachandran and Anstis, 1986; Bruno and Gerbino, 1991; Lorenceau and Alais, 2001). The integration of local motion signals into a larger boundary may prevent local motion from being seen (Shipley and Kellman, 1997). Consistent with this idea, some evidence suggests that when element transformation events can be interpreted as occlusions, apparent motion is suppressed (Sigman and Rock, 1974; Holcombe, 2003; Ekroll and Borzikowsky, 2010). Outside of the integration range, however, the percept reverts to inter-element apparent motion. Since at least two non-collinear signals (i.e., three non-collinear elements) are needed to define an edge, inter-element apparent between the first two elements (the first motion signal), is suppressed only after the third element disappears and a second motion signal is generated.

Finally, an important feature of these experiments is that they bring to bear both subjective and objective methods for evaluating illusory contour perception. Each has potential advantages and disadvantages. SBF is a perceptual phenomenon: what observers see matters. But whereas we assume that perceptual reports convey information about what is seen, they also potentially reflect many other factors, including variations in criteria or use of scales by participants, their understanding of instructions, and possibly their hypotheses about what the experimenter is looking for. Objective paradigms, on the other hand, in which participants' performance can be compared to an objectively correct answer, may more readily avoid some criterion issues, and can be more revealing about underlying mechanisms, but only if the task really depends on the relevant perceptual representations. If there are other strategies for succeeding at a task, all bets are off. In the present work, the concordance of the spatial and temporal parameters that produce clear perception of contours with the parameters that allow accurate performance supports the idea that the methods are converging on contour perception through SBF.

### Potential mechanisms of SBF: motion energy filters as spatiotemporal edge detectors

In the present experiments, we have demonstrated that SBF can occur when the virtual object is a single, small edge or bar. In two-stage model of SBF, these local edges are the fundamental units from which larger, global shapes are constructed (Shipley and Kellman, 1994, 1997; Erlikhman and Kellman, 2015). Formally, it was previously shown that only two velocity vectors between successively transforming, non-collinear elements are sufficient to unambiguously determine the orientation and speed of the perceived edge. Here, we consider how these signals may be extracted and combined to produce SBF under certain conditions and apparent motion in others.

We offer the conjecture that local edge orientations are detected from successive element transformation events by responses of motion energy filters (van Santen and Sperling, 1984; Adelson and Bergen, 1985; Watson and Ahumada, 1985). First-order motion filters detect changes in luminance contrast over space and time. Second-order filters detect contrast differences over time (Chubb and Sperling, 1988, 1989). Since SBF can occur with a variety of element transformations, some of which would not signal any consistent motion energy for first-order filters (Shipley and Kellman, 1994), this conjecture involves both first-order and second-order motion energy filters. For simplicity, in the discussion that follows, we use cases that involve first-order motion.

At first glance, this conjecture seems problematic, in more than one way. Sequential offsets and onsets of elements in SBF would produce responses of moving contrast signaling the motion of dots along the sawtooth pattern. In other words, element-to-element apparent motion should be seen, not a larger, laterally moving edge fragment. This is exactly what is predicted by motion energy models (Adelson and Bergen, 1985; Challinor and Mather, 2010). These local motion signals along the sawtooth do not match either the orientation or the motion direction of the bar. Furthermore, simply by changing the temporal parameters without altering element positions (Movie 4), which would correspond to changing the magnitude, but not direction of these velocity vectors, it is possible to change the perceived tilt of the bar. This too cannot be predicted from a single filter. Most importantly, however, motion energy filters are usually applied to stimuli in which oriented contrast is given in each frame. As such, the spatial orientation profiles of relevant filters are determined at the outset, and what gets determined from sets of activated filters is motion direction and velocity. In SBF, not only must the velocity be determined, but the edge itself must be recovered.

What we are suggesting here is that these same filters serve a separate function when oriented contrast is not given at any moment in the spatial array of the stimulus. In SBF, no frame contains contrast oriented preferentially along the edge fragments that end up being perceived. Under these conditions, the dual function of motion energy filters becomes more observable, as responses of populations of filters having different characteristic spatial orientation profiles may allow specification of local perceived edge orientations.

**Figure 9:**
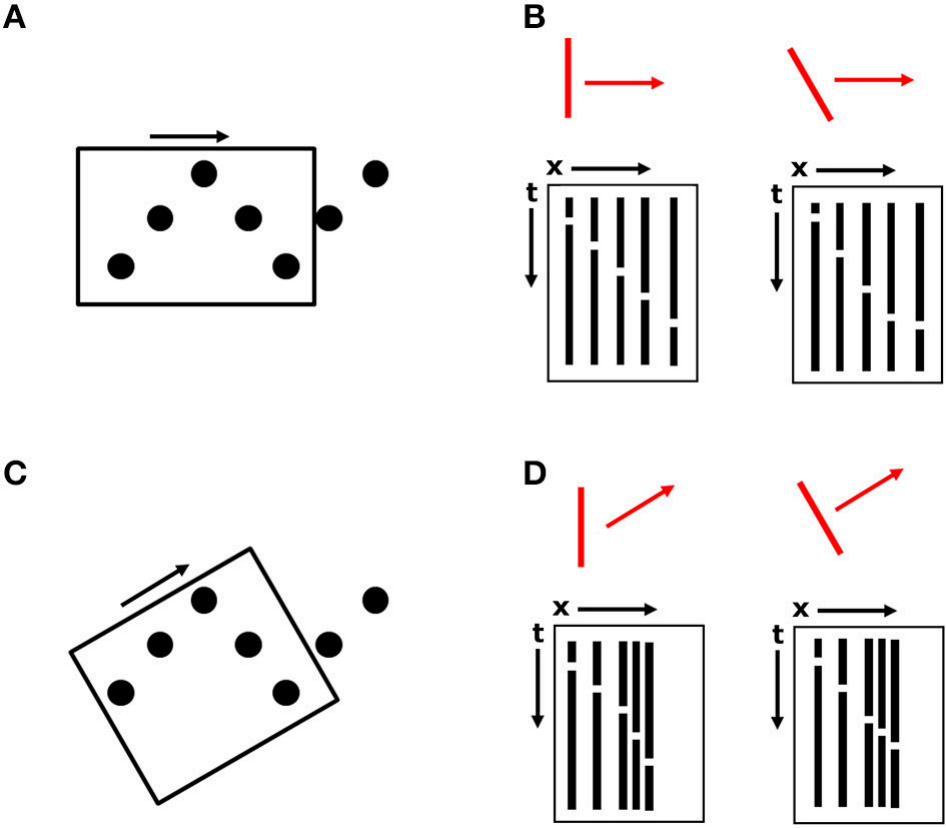
**An illustration of large motion detectors spanning the height of the sawtooth pattern. (A)** A motion detector with a horizontal preferred orientation, indicated by the orientation of the rectangle and the black arrow. **(B)** Space-time diagrams for the two oriented bar stimuli, indicated in red, collapsed along the y-dimension. **(C)** A second, overlapping motion detector with a different preferred orientation. **(D)** Space-time diagrams for the motion detector in **(C)**.

The key to this proposal, relevant to the current displays, is that larger motion energy filters may coexist with those that would specify local element motion along the sawtooth pattern. In Figure 9A, a larger spatiotemporal filter whose receptive field encompasses most or all of the height of the sawtooth display is depicted by a rectangle with its orientation given by the sides of the box and the small arrow indicating its preferred motion direction (i.e., horizontal). In the current experiments, the maximum height of the sawtooth pattern was 4°, well within the range of estimated motion detector receptive field sizes (Anderson and Burr, 1987, 1989, 1991). For the detector shown in Figure 9A, which has a spatial orientation sensitivity for a vertical orientation along with sensitivity to horizontal motion, two space-time diagrams are shown in Figure 9B depicting the sequential disappearances and reappearances of the five elements within the motion detector's receptive field, ignoring the y-dimension, in response to two different stimuli, shown in red. The five columns correspond to the five stationary dots. White breaks in the columns indicate times when a dot disappears and becomes invisible. Although the space-time diagram does not correspond to continuous motion, such detectors would still be activated (Adelson and Bergen, 1985).

For a vertically oriented bar stimulus (Figure 9B, left diagram), the time between element disappearances and reappearances is constant, as in Experiments 1 and 2. This is indicated by the fact that the white breaks in the black columns of the space-time diagram lie on a straight line. If elements are disappearing and reappearing at a constant rate, then either the bar is vertical and moving at a constant velocity, or the bar is tilted and changing velocity between elements. For a tilted bar moving at a constant velocity (right diagram), the time between transformation events is longer for elements lying on paths orthogonal to the orientation of the bar and is shorter for elements lying on paths parallel to the bar.

If this single detector were the only detector activated, then understanding the perceptual output of a moving, vertically oriented bar would be straightforward. Unfortunately, the visual system needs to solve certain ambiguity and aperture problems to combine or adjudicate the responses of multiple such detectors. An example of another, spatially overlapping detector is shown in Figure 9C. Space-time diagrams are also shown for this detector, with the x-axis of the diagram corresponding to the long axis of the detector (its preferred motion direction). This detector would also be activated to some extent by both vertical and tilted bars. Therefore, while Figure 9 indicates that motion energy filters selective for spatial orientation and motion direction could potentially be used for recovery of oriented edge fragments in SBF (despite the absence of spatially oriented contrast in any frame), it also illustrates that there is no single detector that is going to simply indicate the “correct” motion. Here, as in other applications of motion energy filter responses, there are ambiguity and/or aperture problems. Several models exist that combine motion vectors into global percepts (e.g., Weiss et al., 2002), but SBF displays pose some additional ambiguities (Shipley and Kellman, 1994; Prophet et al., 2001). First, the output of such motion models is typically a global motion signal, not an edge orientation. Second, the motion signals that are integrated appear in different regions of the visual field, but concurrently; in SBF, when only a single element transforms per frame, input signals accrue one at a time. Third, orientation information of an object's boundary may often be explicit in the image, but in SBF displays when the elements are circles, no edge orientation information about the virtual object is available in a static image. This describes a nefarious type of aperture problem that exists in SBF, which has elsewhere been called the “point-aperture problem” (Prophet et al., 2001). Unlike the classic aperture problem in which concurrently available motion vectors orthogonal to moving edges are integrated across several small apertures (Wallach, 1935; Adelson and Movshon, 1982; Nakayama and Silverman, 1988a,b; Shimojo et al., 1989), in SBF, in which each element may itself be treated as a small aperture, there are no edges to which the motion vectors are orthogonal, and their direction is therefore ambiguous.

Thus, our conjecture about larger motion energy filters having a dual function of specifying not only motion but spatial orientation in sparse displays will require elaboration of the solutions to several kinds of ambiguity problems. One is the adjudication of outputs specifying local element motion vs. larger edge orientations. It has been previously observed that local element motions in SBF (even when the element transformation used is a local displacement) get co-opted into the perception of a larger, coherent, moving figure (Shipley and Kellman, 1994), but the mechanisms for this remain undetermined. A second ambiguity issue has two parts: A single detector has a combination of a preferred spatial orientation and (orthogonal) motion direction (thus, when activated, it can be thought of as signaling that orientation and direction). Note that, as shown in Figure 9B, two differently oriented virtual bars in SBF will both activate the single detector in Figure 9A. In other words, both a vertical and oblique virtual edge in the stimulus will activate this detector to signal a vertical edge. Conversely, both the moving, virtual vertical and oblique edges also activate the detector sensitive to oblique orientation in Figure 9C. Thus, a given detector responds to different stimulus orientations and a given orientation in the stimulus activates detectors that signal multiple orientations. For both of these related ambiguities, we believe that a constant velocity constraint may provide the basis of a solution. For the two possible stimuli shown in Figure 9B, only the vertical stimulus orientation produces a constant velocity in the space-time diagram. Similarly, only the oblique stimulus orientation produces a constant velocity in the oblique detector output. A constant velocity over short time spans seems to be a sensible ecological constraint and exists in several motion models (e.g., Johnston et al., 1992; Note, however, that our idea of what “correct” encoding of the virtual stimulus here also incorporates a constant velocity constraint in our construction of the displays. An oblique virtual edge that changed velocity as given in the response profile in Figure 9B would be exactly the stimulus). Finally, if various motion/orientation signals can be distilled via suitable constraints into a local edge orientation of constant velocity, these fragments still suffer from the classical aperture problem of an oriented moving, even constant velocity (Shipley and Kellman, 1994).

Further work is needed to determine methods by which the visual system may combine information from populations of motion/orientation detectors in SBF displays. Another high priority in evaluating our conjecture about motion energy filters involves tying information about spatial and temporal receptive field sizes of motion energy filters to limitations on the conditions under which SBF edge formation occurs. In the experiments reported here, the relatively sharp confinement of edge formation effects to a temporal window of about 165 ms in which a set of three element transformations must occur suggests a fixed mechanism, rather than some more open-ended perceptual inference. It should be possible to relate limits on SBF directly to properties of spatiotemporal filters in the visual system.

Finally, we consider the role of pursuit in these displays and its relation to the proposed filters. We found in pilot work that if observers fixate at any point on the sawtooth, the percept reverts to that of apparent motion. We therefore emphasized to subjects to attempt to track the sequence of element changes. This observation suggests that the displays can be inverted so that the bar is stationary, the observer fixates on the bar, and the pattern of elements moves behind it. An illusory bar is indeed seen in such displays. We had opted to use stationary elements and a moving bar in these experiments because this more closely matches previous work using SBF. Events in typical SBF displays are spatiotemporally dense, so that smooth pursuit eye movements are not necessary (Shipley and Kellman, 1993a, 1994, 1997). In fact, one cannot focus on all parts of the SBF-defined boundaries at the same time, especially when the objects are large. However, in the limiting cases when there are relatively few events—a single event every few frames—tracking is necessary. We plan on carrying out studies with stationary, SBF-defined figures and moving elements in future studies.

Nevertheless, one may ask how and why a motion energy filter would respond to such displays that contain a stationary, illusory edge defined by the transformations of moving elements. Our conjecture is that these filters are spatiotemporal orientation filters and that they serve a dual role and signal a dual output. As such, they may be activated by temporally extended inputs that signal an oriented edge whose motion is zero. It is important to remember that these displays do not contain an illusory moving or stationary bar, only element transformation events. The visual system is not tracking a bar or detecting its motion directly. Rather, these spatiotemporal energy filters are accumulating signals from each event. Tracking is necessary when signals are sparse in order to accumulate enough positionally appropriate events within the receptive field of the detector. This requirement is easily satisfied for a stationary bar and a fixating observer. For a moving bar, if the observer fixates, dot disappearance and reappearance events would occur far apart from one another, falling on disparate parts of the retina, and therefore would not stimulate the same detector.

Illusory contours in displays with a stationary bar and moving elements could also be accounted for by a motion contrast model since there are clearly separable regions of moving elements and regions in which no elements are moving (Tadin et al., 2003). Note that such a display is identical on the retina to what occurs in the current experiments when observers track the empty region where the illusory bar is seen. A motion contrast account could therefore apply to these displays as well. However, tracking is not necessary to perceive illusory contours in SBF displays in general, particularly when they contain dense arrays of elements. It is not clear how a motion contrast model would account for displays in which observers are fixating and the elements are stationary, particularly when element transformation events are rotations, shape-changes, or position changes in which no central object region is clearly defined (Shipley and Kellman, 1993a, 1994).

If this conjecture about motion energy filters in SBF is correct, it suggests that these filters have a dual function, one that is not readily discernible when stimulus orientation is explicitly given by contrast: they are also edge filters. Moreover, on this hypothesis, SBF is not an esoteric perceptual illusion, but is actually more indicative of basic processes implemented across the visual field for extraction of edges, motion, and their interaction. Of course, many details of this conjectured edge-motion duality in basic visual filtering remain to be worked out.

## Author contributions

GE and PK jointly designed the experiments. The experiments were programmed and conducted by GE. Data were analyzed by GE. Manuscript written jointly by GE and PK.

## Author note

Portions of this work were presented at the 15th Annual Meeting of the Vision Sciences Society, May 2015. Portions of this work were submitted in the partial fulfillment of the requirements for the degree of Doctor of Philosophy in Cognitive Psychology for GE.

### Conflict of interest statement

The authors declare that the research was conducted in the absence of any commercial or financial relationships that could be construed as a potential conflict of interest.

## Supplementary material

Movie 1 **A square defined by SBF**. On each frame, elements either disappear (become black) when entering the boundary of the square or reappear (become white) when exiting the boundary. The elements transform quantally, that is, all at once.

Movie 2 **A sawtooth stimulus similar to the kind used in Experiments 1 and 2**. Apparent motion is seen: a white dot appears to move along the sawtooth path.

Movie 3 **The same stimulus as in Movie 2, except with altered temporal parameters**. If one tracks the element transformations laterally as they occur, one can see a thin, white, illusory bar spanning the height of the sawtooth pattern. This movie is best seen in the dark, some distance away from your monitor.

Movie 4 **The same stimulus as in Movies 2, 3**. The only difference is that the temporal properties are not constant between element transformations. The resulting percept is of a tilted moving bar.
